# A cross-sectional study on vitamin D deficiency and cardiac electrophysiology: Evaluating the frontal QRS-T angle

**DOI:** 10.1097/MD.0000000000042694

**Published:** 2025-05-30

**Authors:** Ezgi Akyildiz Tezcan, Hüseyin Tezcan

**Affiliations:** a Department of Physical Medicine and Rehabilitation, Selcuk University Faculty of Medicine, Konya, Turkey; b Department of Cardiology, Selcuk University Faculty of Medicine, Konya, Turkey.

**Keywords:** arrhythmia, cardiovascular health, electrocardiogram, frontal QRS-T angle, vitamin D deficiency

## Abstract

Vitamin D deficiency is a global health issue, primarily recognized for its role in bone health and calcium absorption. Emerging research has expanded its significance to cardiovascular health. This study aimed to assess the influence of vitamin D deficiency on the frontal QRS-T (f[QRS-T]) angle on electrocardiograms, a potential marker of arrhythmic risk and cardiac mortality, to address a notable gap in the current understanding. In this prospective cross-sectional study, 357 individuals were assessed, with 75 excluded (60 for not meeting criteria, 10 declined, 5 other reasons). A total of 282 participants were grouped by vitamin D levels: vitamin D-deficient (<20 ng/mL, n = 185) and non-deficient (≥20 ng/mL, n = 97). After excluding 7 participants for incomplete tests, data from 275 (deficient: n = 180; non-deficient: n = 95) were analyzed. Electrocardiograms were evaluated by a blinded cardiologist, and data were analyzed with appropriate statistical methods. Our analysis revealed no significant differences in the demographic characteristics, laboratory findings, and f(QRS-T) angle between the groups with and without vitamin D deficiency. This study indicated that vitamin D deficiency does not significantly affect the f(QRS-T) angle in our sample, suggesting a limited role of vitamin D status in the risk of ventricular arrhythmias. These findings contribute to the discussion on the role of vitamin D in cardiovascular health and underscore the need for further studies to guide clinical decisions regarding vitamin D supplementation and cardiovascular risk management.

## 1. Introduction

Vitamin D deficiency, affecting an estimated 1 billion individuals globally, is primarily known for its role in calcium absorption and bone health.^[1]^ However, recent research has expanded its significance to encompass a variety of physiological processes, such as cell differentiation, immunomodulation, and hormonal system regulation, underscoring its broader impact on overall health and disease prevention.^[2]^

The biologically active form, 1,25-dihydroxyvitamin D, operates through the vitamin D receptor (VDR) and, influences gene transcription responsible for various biological responses.^[2]^ Recent studies have revealed the presence of VDR and 1α-hydroxylase in cardiomyocytes, endothelial cells, and vascular smooth muscle cells, linking vitamin D to cardiovascular health and prompting further exploration.^[1]^

Observational human studies have consistently reported an inverse correlation between vitamin D status and cardiovascular risk factors.^[1]^ However, the landscape is nuanced, with contradictory evidence from large-scale studies such as Mendelian randomization and recent trials such as the Vitamin D Assessment trial and the Vitamin D and Omega 3 Trial, complicating the relationship between vitamin D supplementation and cardiovascular outcomes.^[3–6]^

In the realm of cardiac diagnostics, electrocardiography (ECG) is an indispensable tool, valued for its cost-effectiveness, noninvasiveness, and prompt availability of results. Within this context, a novel metric, the frontal QRS-T (f[QRS-T]) angle, has emerged to quantify the angular disparity between the QRS complex and the- T-wave axes on an ECG. Beyond its technical simplicity, the f(QRS-T) angle is clinically significant because of its association with an elevated risk of arrhythmias, all-cause mortality, and sudden cardiac death.^[7]^

This study sought to bridge these gaps by investigating the potential influence of vitamin D deficiency on the f(QRS-T) angle. By examining individuals with varying vitamin D statuses, we aimed to clarify the relationship between vitamin D, cardiovascular health, and the risk of specific cardiac events. Our study contributes additional information to the existing literature and may help guide future research and clinical practice regarding vitamin D and cardiovascular health.

## 2. Materials and methods

### 2.1. Study design and participants

This prospective cross-sectional study was approved by the local ethics committee of the medical faculty and, adhered to the guidelines outlined in the World Medical Association Declaration of Helsinki (2000). All participants provided written informed consent prior to enrollment.

The investigation involved individuals aged 18 years and above seeking services at a physical medicine and rehabilitation outpatient clinic between September 2023 and December 2023. Exclusion criteria included a history of specific cardiac conditions, such, as, coronary artery disease, dysrhythmia, heart valve disease, hypertension, and heart failure. The exclusion criteria were pregnancy, malignancy, major kidney or liver disease, uncontrolled diabetes mellitus, hypothyroidism, or other severe systemic disorders. Participants with vitamin D levels below 20 ng/mL were classified as vitamin D deficient, while those with higher levels served as the control group, in accordance with the Endocrine Society Clinical Practice Guidelines and Institute of Medicine.^[8]^ Demographic and clinical characteristics of the participants were recorded systematically. No changes to the methods were made after the trial commenced.

### 2.2. Electrocardiogram Examination

**Figure 1.**
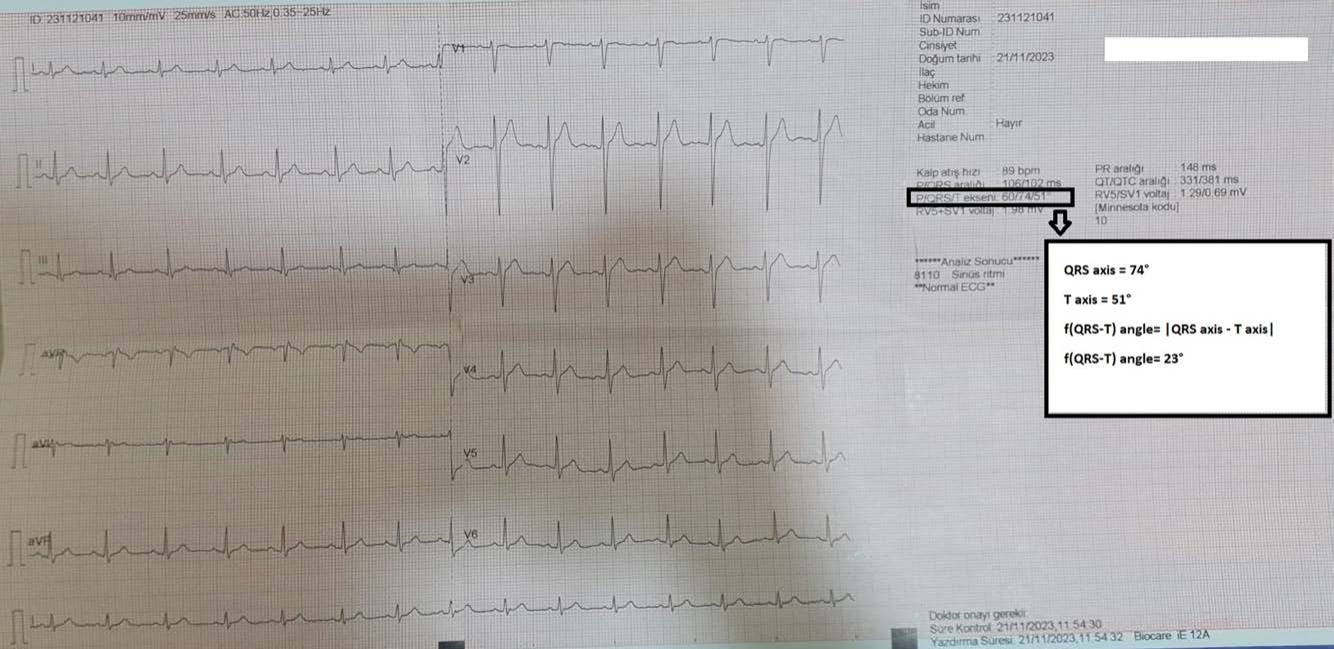
Measurement of the frontal QRS-T angle on a standard 12-lead ECG. ECG = electrocardiogram, QRS-T = QRS complex and T wave.

All patients underwent resting 12-lead ECG recording using a Biocare IE12A machine (Shenzhen Biocare Biomedical Equipment, China). During this procedure, the patients were placed in the supine position. ECG was performed with specific settings: speed, 25 mm/s; amplitude, 10 mm/mV; and filter range 0.16, 100 Hz. A blinded cardiologist performed ECG analysis by focusing on several key parameters. The f(QRS-T) angle was determined as the absolute difference between the frontal plane QRS axis and the frontal plane T axis, with angles exceeding 180° calculated by subtracting from 360°^[7]^ (Fig. 1). In addition to the f(QRS-T) angle, the PR and QRS durations were measured, which were defined as the interval from the onset of the P-wave to the beginning of the QRS complex and the length of time from the start to the end of the QRS complex, respectively. Heart rate was calculated using a standard method of counting the number of QRS complexes in a 10-seconds ECG strip and multiplying by 6 to obtain the rate per minute.

### 2.3. Statistical analyses

Statistical analyses were performed using SPSS version 22.0 (IBM, Armonk, New York). Categorical variables were expressed as numbers and frequencies, whereas continuous variables were described as mean (standard deviation) and median (interquartile range). The chi-square test was used to compare the categorical variables. Normal distribution was evaluated both visually (histograms) and analytically (Kolmogorov–Smirnov). Mann–Whitney *U* and independent *t*-tests were applied for non-normally and normally distributed data, respectively. Significance was set at *P* < .05, and a 95% confidence interval was used for the interpretation of results.

## 3. Results

A total of 357 individuals were assessed for eligibility. Of these, 75 were excluded due to not meeting inclusion criteria (n = 60), declining to participate (n = 10), or other reasons (n = 5).

### 3.1. Total of 282 participants were categorized into 2 groups

Vitamin D-deficient group: 185 participants (180 completed the study, while 3 were lost to follow-up, and 2 did not complete ECG or lab tests).

Non-deficient group: 97 participants (95 completed the study, while 2 did not complete ECG or lab tests).

Data from 275 participants (vitamin D-deficient: n = 180; non-deficient: n = 95) were included in the final analysis.

The majority of the participants in our study cohort were females, constituting 85.81% of the total. There were no differences between the groups in terms of age or body mass index (*P* = .708 and, *P* = .107, respectively).

PTH, albumin-corrected calcium, and phosphorus levels were compared between the groups because of their relevance to the vitamin D metabolic pathways. Albumin-corrected calcium was calculated using the following equation: albumin-corrected calcium (mg/dL) = serum total calcium (mg/dL) + 0.8 × [4.0 − serum albumin (g/dL)].^[9]^ No significant differences were observed in PTH, albumin-corrected calcium, or phosphorus levels between the 2 groups (median (IQR) 46.4 (35.95–65.32), 46 (32.4–56.4), *P* = .173; mean (SD) 9.15 (0.34), 9.18 (0.33); *P* = .489; mean (SD) 3.28 (0.55), 3.37 (0.63), *P* = .251). Additional detailed information on demographic and other laboratory variables is presented in Table 1.

**Table 1:** Demographic and clinical findings of participants.

Characteristic	Vitamin D-deficient (<20 ng/mL; n = 180)	Vitamin D non-deficient (≥20 ng/mL; n = 95)	*P* value
Age (yr)	42.34 (10.96)	42.95 (12.79)	.708
BMI (kg/m^2^)	28.28 (5.29)	27.04 (5.99)	.107
Gender, Female; n (%)	159 (88%)	77 (81%)	.143
Smoking status; Smoker n (%)	42 (23.3%)	25 (26.3%)	.689
Hemoglobin (g/dL)	13.25 (12.33–14.10)	13.2 (12.4–14.4)	.504
WBC (10^9^/L)	7.38 (6.18–8.66)	7.42 (6.21–8.44)	.918
Platelet (10^9^/L)	278 (238–326)	263 (229–320)	.281
CRP (mg/dL)	3.3 (1.43–5.18)	3.1 (1.5–5.2)	.701
ESR (mm/h)	9 (5–14)	11 (6–15)	.120
AST (U/L)	18 (15.25–23)	19 (16–23)	.351
ALT (U/L)	17 (13–23.75)	18 (13–26)	.206
Creatinine (mg/dL)	0.7 (0.63–0.8)	0.72 (0.66–0.82)	.155

ALT = alanine transaminase, AST = aspartate transaminase, BMI = body mass index, CRP = C-reactive protein, dL = deciliter, kg = kilogram, L = liter, m^2^ = square meter, mg = milligram, N = number, WBC = white blood cells.

No statistically significant difference in the f(QRS-T) angle was observed between the 2 groups (median (IQR): 18 (8–33.2), 19 (10–34); *P* = .358). Additionally, heart rate was assessed, with a mean (SD) of 74 (9) beats/min in the vitamin D-deficient group and 72 (8) beats/min in the non-deficient group, which did not reach statistical significance (*P* = .294). Further ECG parameters, such as PR interval and QRS duration, also did not show significant differences (PR interval mean (SD) 160 (20) ms vs 158 (18) ms, *P* = .423; QRS duration median (IQR) 90 (82–98) ms vs 88 (80–96) ms, *P* = .337).

## 4. Discussion

In our study, no significant difference was observed in the f(QRS-T) angle between individuals with and without vitamin D deficiency. These findings support the notion that vitamin D deficiency may not be a significant contributor to the elevated risk of ventricular arrhythmias observed in our study cohort.

The interplay between vitamin D levels and cardiovascular outcomes has been a subject of extensive research. Observational studies have often posited a connection between lower vitamin D levels and a range of cardiovascular conditions, including hypertension, atherosclerosis, and heart failure.^[1]^ However, the consistency of these associations has not yet been investigated in interventional studies. Notably, large-scale and robust studies, such as the Vitamin D Assessment and Vitamin D and Omega 3 Trial, have failed to demonstrate a significant benefit of vitamin D supplementation in the prevention of cardiovascular diseases.^[4–6]^ This apparent discrepancy between observational and interventional studies underscores the intricacies inherent in the relationship between vitamin D levels and cardiovascular health.

Potential mechanisms by which vitamin D deficiency could influence cardiac events include alterations in calcium-handling proteins and ion channels via the VDR, which is expressed in cardiomyocytes. Recent pediatric research by Guzelcicek et al found that lower vitamin D status correlates with changes in the Index of Cardio-Electrophysiological Balance, indicating that deficient levels of vitamin D might predispose individuals to arrhythmic risks by affecting cellular repolarization.^[10]^ Additionally, Vanga et al reviewed the broader role of vitamin D in cardiovascular health and proposed that deficiency might exacerbate endothelial dysfunction, increase inflammatory pathways, and influence cardiac remodeling, each of which can contribute to arrhythmogenesis and adverse cardiovascular outcomes.^[11]^ While our findings did not reveal significant differences in the f(QRS-T) angle, these mechanistic insights highlight the complexity of vitamin D’s role in cardiac physiology and underscore the need for further research to determine which subgroups might be more affected.

Our study provides valuable insights into this complex field, especially given the scarcity of studies examining the association between vitamin D and arrhythmias using specific ECG parameters. Previous studies, including those by Anees et al and Canpolat et al, predominantly focused on atrial arrhythmias in relation to vitamin D deficiency. However, these studies varied in patient demographics and methodologies, thereby limiting their direct comparability with our research.^[12,13]^ For instance, Anees et al identified correlations between lower vitamin D levels and prolonged P-wave and PR durations, but their study was characterized by an older demographic and included hypertensive participants, which significantly differs from our study population.^[12]^ Canpolat et al noted an increase in P-wave dispersion in vitamin D-deficient patients; however, no changes were observed after replacement therapy.^[13]^

Several studies on ventricular arrhythmias are particularly noteworthy. Zhang et al found no correlation between 25-hydroxyvitamin D concentration and QT interval.^[14]^ This extensive study included a thorough cross-sectional analysis of the Third National Health and Nutrition Examination Survey III and the Atherosclerosis Risk in Communities study, encompassing 7312 patients.^[14]^ The findings of this large-scale study are consistent with our results.^[14]^ Conversely, the 2014 study by Tuliani et al on 5108 individuals from the Third National Health and Nutrition Examination Survey III indicated an association between vitamin D deficiency and major ECG abnormalities.^[15]^ They reported that major ECG abnormalities in individuals with 25-OH vitamin D levels ≤ 40 ng/mL were independently and significantly associated with an increased risk of long-term, all-cause composite cardiovascular events, cardiovascular events, and ischemic heart disease.^[15]^ There are significant differences between their study and ours, particularly in participant age, sex distribution, and inclusion of individuals with hypertension.^[15]^ A critical distinction is the use of a 40 ng/mL threshold for vitamin D levels to determine outcomes.^[15]^ In our study, only 2 of 275 patients had vitamin D levels exceeding this threshold, rendering the impact on cardiovascular events at higher vitamin D levels uncertain.

Furthermore, a 2017 study by Nalbant et al and a 2020 study by Ravichandran et al in patients with type 2 diabetes mellitus did not find significant differences in corrected QT intervals based on vitamin D levels, which is consistent with our study.^[16,17]^ Additionally, a recent randomized controlled study in 2023 revealed that vitamin D supplementation did not significantly alter various ECG parameters, although a modest but significant difference was noted in Cornell voltage.^[18]^ While these findings partially coincide with our research, the differences in study populations, particularly concerning average age, sex distribution, and inclusion of patients with hypertension, are significant.

Our study has several limitations that should be acknowledged. The single-center, observational design and relatively small sample size limit the generalizability of our findings. Additionally, the cross-sectional nature of the study prevents the establishment of causal relationships between vitamin D deficiency and cardiac electrophysiological parameters. Variability in defining vitamin D deficiency thresholds and the lack of data on seasonal variations or sun exposure further complicate the interpretation of our results. Despite these constraints, our findings provide valuable insights and underscore the need for larger, multicenter studies to validate and expand upon this research.

## 5. Conclusion

In conclusion, our study adds to the existing research on vitamin D and its potential relationship to ventricular arrhythmias. Despite our findings suggesting no significant impact of vitamin D deficiency on the f(QRS-T) angle, the complexity and diversity of existing research underscores the need for further in-depth, multicenter, and longitudinal studies. These future investigations are crucial for clarifying the role of vitamin D in cardiovascular health and potentially guiding clinical practices regarding vitamin D supplementation and monitoring.

## Author contributions

**Conceptualization:** Ezgi Akyildiz Tezcan, Hüseyin Tezcan.

**Data curation:** Ezgi Akyildiz Tezcan, Hüseyin Tezcan.

**Formal analysis:** Ezgi Akyildiz Tezcan, Hüseyin Tezcan.

**Funding acquisition:** Ezgi Akyildiz Tezcan, Hüseyin Tezcan.

**Investigation:** Ezgi Akyildiz Tezcan, Hüseyin Tezcan.

**Methodology:** Ezgi Akyildiz Tezcan, Hüseyin Tezcan.

**Project administration:** Ezgi Akyildiz Tezcan, Hüseyin Tezcan.

**Resources:** Ezgi Akyildiz Tezcan, Hüseyin Tezcan.

**Software:** Ezgi Akyildiz Tezcan, Hüseyin Tezcan.

**Supervision:** Ezgi Akyildiz Tezcan, Hüseyin Tezcan.

**Validation:** Ezgi Akyildiz Tezcan, Hüseyin Tezcan.

**Visualization:** Ezgi Akyildiz Tezcan, Hüseyin Tezcan.

**Writing – original draft:** Ezgi Akyildiz Tezcan, Hüseyin Tezcan.

**Writing – review & editing:** Ezgi Akyildiz Tezcan, Hüseyin Tezcan.
